# Psychiatric disorders and holistic therapies

**DOI:** 10.4103/0973-6131.72627

**Published:** 2010

**Authors:** Thaiyar M Srinivasan

**Affiliations:** Swami Vivekananda Yoga Anusandhana Samsthana (A Yoga University), No.9, Appajappa Agrahara Chamarajpet, Bangalore - 560 018, India. E-mail: tmsrini@gmail.com

Psychiatric disorders are the most prevalent problems of modern living, with drugs for the management of these disorders selling in billions of dollars every year around the world. It is estimated that about 50 million people have either moderate or severe forms of psychiatric disorders in India.[1] The same article mentions that India provides one bed per 40,000 population and three psychiatrists per million population. This works out to approximately 3000 trained psychiatrists for the entire country. The expertise is available mostly in the cities and not for the rural population, wherein the requirements for psychiatric support and management are critical. The rural group can ill afford loss of workdays, hospital admissions, costly tests, and other requirements of modern medicine.

It is believed that over 46 per cent of the U.S. population might be affected with at least one psychiatric disorder and at least a quarter of the population of that country have two or more disorders [3, p. 1]. This is a shocking revelation; unfortunately, many industrially advanced countries reveal similar numbers. Although the Indian statistics are not as threatening, nonetheless, the prevalence of psychiatric disorders enormously outstrip the available resources. Hence, there is an urgent need for both prevention and non-pharmacological management of psychiatric disorders.

There are many non-pharmacological methods for the management of psychiatric problems. Biofeedback and Yoga are emerging as two specific techniques that seem to help these patients. In the biofeedback procedures, instantaneous information regarding a physiological variable (such as heart rate) is provided to the client and the client learns to control the parameter using the desirable methods. In the example of the heart rate, the patient may be asked to reduce the heart rate through ‘appropriate thinking’ and he/she learns to do so with some hours of training. Similarly, skin temperature, sweating, blood pressure, electromyographic activity, Heart Rate Variability (HRV), and so on, can be controlled if the person is made aware of them through proper instrumentation.

Learning to control physiological functions that are normally autonomic seem to have many advantages. There is information transfer from the periphery to the center; for example, learning to control the temperature of a finger sends strong signals to the brain to reset some of the physiological functions within limits. The heart sends signals to the brain through many ways: through hormones, through its electrical activity, through vagus nerve, and through blood pressure waves.[2] The Mind–Body complex can be made to go through a relaxed, hypometabolic, parasympathetic dominance. Such a change in physiology results in reduced stress and anxiety, reduces cortisol levels, increases DHEA or dehydroepiandrosterone, and improves the functioning of the prefrontal cortex, enhancing mental clarity. A client, who has learned the biofeedback procedure, can control the physiological function even without a feedback instrument; this gives confidence to the person and the ability to master one’s own physiology, within normal limits.

Al present, some biofeedback types seem to be useful, while others are still in the experimental stages. Neurofeedback for Attention Deficit Hyperactivity Disorder (ADHD) has been around for almost 15 years with a positive response from the clients. Here the electroencephalogram (EEG) of the person is displayed as a game module. The person is trained to increase beta waves under normal, eyes-open condition, and there are clinical improvements in the ADHD of the clients. Biofeedback is also seen to enhance memory, improve sleep in insomnia patients, as also depression and PTSD (Post Traumatic Stress Disorder). Although it usually takes a long time for the client to train in biofeedback procedures, it has no known side effects, trauma or negative emotional responses. It is not known whether the changes seen are long lasting; however, short-term gains are observed in both the clinical and personal outcomes.

Yoga is another method for the management of psychiatric disorders. A recent book, written by a dedicated Kundalini Yoga teacher and an avid researcher, has a wealth of information about psychiatric disorders, especially those with multiple problems.[3] An earlier book by the same author deals with specific psychiatric disorders including obsessive-compulsive disorder, acute stress disorder, PSTD, ADD/ADHD and other psychiatric problems.[4] In the recent book,[3] a detailed summary of the latest research in many psychiatric problems has been brought out and the difficulty in the management of these problems through drugs alone is summarized from the published literature. Each chapter then provides detailed Kundalini Yoga practices for specific disorders, followed by case studies and results, as narrated by the patients themselves. It is undoubtedly a well-researched book, both on the pharmacological intervention of psychiatric disorders (which is complex with many side effects) and Kundalini Yoga methods. There is no doubt that Yoga and meditation can provide an excellent alternative for the treatment of psychiatric disorders.

It is necessary for Indian Yoga Research Institutes to look into this alternative closely and provide an integrated approach to many psychiatric problems. There are a few articles by Indian researchers on the effect of Yoga on psychiatric disorders.[5,6] However, more work is needed to move this from the experimental to a clinic setting. After all, if citta could be brought under control, it is likely all else follows. Mental poise, biochemical homeostasis, neurological normalcy followed by *karmasu kausalam* or skill in action would be possible.
